# Healing of Alveolar Sockets Treated with Concentrated Growth Factors: A Split-Mouth Study

**DOI:** 10.3390/ma15144859

**Published:** 2022-07-12

**Authors:** Marco Mozzati, Margherita Tumedei, Giorgia Gallesio, Giulio Menicucci, Carlo Manzella, Tiziano Testori, Massimo Del Fabbro

**Affiliations:** 1 SIOM Oral Surgery and Implantology Center, 10126 Turin, Italy; marcomozzati@siompoliambulatorio.it (M.M.); giorgiagallesio@siompoliambulatorio.it (G.G.); 2 Department of Biomedical, Surgical and Dental Sciences, Università degli Studi di Milano, 20122 Milan, Italy; margherita.tumedei@unimi.it (M.T.); info@tiziano-testori.it (T.T.); 3 Prosthodontic Department, School of Dentistry, University of Turin, 10124 Turin, Italy; giuliomenicucci@libero.it (G.M.); carlo.manzella@studiosphera.it (C.M.); 4 Dental Clinic, IRCCS Orthopedic Institute Galeazzi, 20161 Milan, Italy; 5 Department of Periodontics and Oral Medicine, School of Dentistry, The University of Michigan, Ann Arbor, MI 48109, USA

**Keywords:** concentrated growth factors, bone regeneration, alveolar socket, postextraction, socket healing, tooth extraction

## Abstract

Background: tooth extraction is a common procedure in oral surgery. The socket healing process involves hard and soft tissues and is characterized by intense remodeling, which may determine consistent dimension changes. Several autologous platelet concentrates (APCs) proved to be effective for enhancing alveolar socket healing after tooth extraction, accelerating socket closure and countering alveolar bone resorption. Concentrated growth factors (CGFs) are one of the most recently developed APCs, and their effect on the socket healing process still needs to be confirmed. Aim: The aim of the present split-mouth study was to evaluate the effectiveness of CGFs in enhancing the healing process in the postextraction alveolar socket and reducing postoperative pain. Methods: One hundred and fifty-four extractions were performed. One of the extraction sockets of each patient was treated with CGFs (test site), and the other socket was unfilled (control site). The main outcomes were: healing index, alveolar dimensions at the crestal level, socket closure, and pain perception. Descriptive statistics of the results were analyzed. Follow-up data were compared to baseline using paired tests. Results: The healing index on day 7 was significantly better (*p* < 0.001) in the test group (5.01 ± 1.30) as compared to the control group (6.65 ± 1.41). The mean visual analog scale for pain (VAS) was significantly higher for the control group when compared to the CGF group in the first 5 days postextraction. There was a trend toward greater socket closure in the CGF group, indicating faster healing, as compared to the control group at 7, 14, and 21 days. Conclusions: CGFs can represent a useful adjunctive tool, considering their mechanical and biological properties, for improving alveolar socket healing and reducing postoperative patient discomfort.

## 1. Introduction

Tooth extraction is one of the most common procedures in oral surgery and is correlated with consistent physiological changes in the alveolar process [1]. The healing process is characterized by different phases: coagulation, inflammation, tissue replacement, and resolution. During the coagulation phase, the platelets release chemokines, growth factors, and matrix components [2]. Moreover, during inflammation, cells are recruited and are able to migrate [3,4]. Subsequently, highly vascularized granulation tissue is produced, which is able to improve tissue oxygenation and nutrient supply. In the following phase, collagen fibrils are synthesized and become progressively organized in the tissue [5]. Trismus, edema, and pain are typical local symptoms in the post-surgical phase and are associated with the augmentation of serum C-reactive protein, fibrinogen, and an increase in oxidative stress. Pain, bleeding, trismus, and swelling may also influence the patient’s postoperative quality of life. For spontaneous wound healing (without using techniques for enhancing the healing process), a long time may be necessary, which increases the risk of complications, such as necrosis, alveolitis, and superinfection, and in some cases, drugs are required to treat pain. The first weeks after the extraction are characterized by intense bone remodeling; this concept has been extensively described in various clinical and pre-clinical studies in the literature [6,7]. The alveolar ridge goes through gradual atrophy, which is more intense in the horizontal (bucco-lingual) dimension compared to the vertical (apico-coronal) dimension and is greater at the vestibular side compared to the lingual/palatal aspect.

Numerous reviews of the literature have reported that autologous platelet concentrates (APCs) are effective for improving alveolar socket healing following tooth extraction.

Among the several APCs, concentrated growth factors (CGFs) can be considered an ideal alternative due to their biological and mechanical properties. CGFs can be applied as a filler material and/or as a covering membrane, and they can be mixed with grafting material, enhancing the cohesion of the graft particles. CGF membranes have a strong mechanical consistency and can be easily sutured [8].

The present split-mouth prospective clinical study aimed to evaluate whether the additional use of CGFs, as compared to the natural healing of postextraction sockets, is beneficial in terms of enhancement of the regenerative process and postextraction alveolar socket preservation.

## 2. Materials and Methods

The IRCCS Istituto Ortopedico Galeazzi Scientific Board, Milan, Italy (No. L2057), approved the protocol. Three private practice surgeons performed all surgeries in accordance with the Helsinki Declaration on research protocols on humans. The inclusion criteria were: the subject required the extraction of at least two teeth due to endodontic issues or periodontal disease, tooth fracture, or non-restorable caries; the subject had to be healthy (ASA-1 or ASA-2 according to the American Society of Anesthesiologists’ classification). The following criteria led to exclusion: teeth with acute infection, subjects smoking more than 10 cigarettes per day, relevant systemic health conditions (ASA-3 or ASA-4), irradiation or chemotherapy to the head or neck region in the 12 months prior to the intervention, pregnancy or lactation, or unwillingness to comply with instructions relative to the follow-up controls. All of the patients enrolled in the present protocol signed an informed consent form and received all information about the surgery and the follow-up.

Seven days before surgery, all patients had a professional oral hygiene session, and three days before the extraction, they started mouth washing with chlorhexidine digluconate 0.2%.

### 2.1. Surgical Procedures

In order to obtain CGFs from each patient at the beginning of the intervention, 9 mL of venous blood was drawn into tubes and placed into a specific centrifuge (Medifuge MF200, Silfradent^®^ Srl, Santa Sofia (FC), Italy). After local anesthesia (mepivacaine 2% with adrenaline 1:100,000), the teeth were extracted without full-thickness flap elevation. For each patient, extractions were performed in the same session. The sockets were carefully debrided, and curettage of the alveolus was performed to remove granulation tissue. The alveolar dimensions at the crestal level were measured with a calibrated probe. In each patient, the test socket was filled with CGFs, and in the control site, the socket was unfilled. No sutures were applied. All of the patients rinsed the surgical wound three times per day for 2 weeks using 0.2% chlorhexidine digluconate and were instructed to have a semiliquid cold diet the day after the extraction.

Antibiotics or analgesics were not routinely prescribed, but the patients could take them in case of need. Each patient was scheduled for three follow-up visits at 7, 14, and 21 days post-intervention. Patients were followed until closure of the socket.

### 2.2. CGF Preparation

CGFs can be considered an evolution of platelet-rich fibrin (PRF), though the protocol for their production has distinctive features. The blood sample is drawn into test tubes that do not contain anticoagulant, and the separation process occurs in one step at a constant temperature but involving consecutive phases with variable rotation speed [9]. This protocol allows 4 fractions to be obtained: the serum, platelet-poor plasma and a fibrin-rich clot, a liquid phase (CGFs) containing growth factors (platelet-rich plasma and fibrin) and white blood cells, and a viscous red clot of erythrocytes and cell fragments (from the top to the bottom of the tube).

### 2.3. Outcome Variables

Healing index: The healing score (Landry, Turnbull, and Howley Index) was evaluated on days 7 and 14 [10,11].

Alveolar dimensions at crestal level: Using a calibrated periodontal probe, the alveolar size was measured horizontally in the vestibulo-palatal/lingual (VP) dimension and in the mesio-distal (MD) dimension. The measurements were taken soon after tooth extraction (baseline) and at each follow-up visit (7, 14, and 21 days post-surgery).

Socket closure: Closure of the alveolar socket at the crestal level was estimated from alveolar size changes and expressed as a percentage relative to the baseline value for both VP and MD dimensions.

Pain: Postoperative pain was assessed daily using the VAS score on a scale from 0 (absence of pain) to 10 (the worst possible pain).

Full-mouth plaque score (FMPS): FMPS is the percentage of tooth sites showing dental plaque relative to the overall number of sites evaluated in the mouth (4 sites per element). This index reflects the degree of oral hygiene of the patient.

Full-mouth bleeding scores (FMBS): FMBS is the percentage of tooth sites that bleed upon slight probing (4 sites per element). This index reflects the degree of gingival inflammation.

### 2.4. Statistical Analysis

The data were summarized by using the mean values and the standard deviation (SD) when the variables were distributed normally. Qualitative variables were described using frequency and percentage. The d’Agostino and Pearson omnibus normality test was used to determine if the data followed a Gaussian distribution. The unpaired Student’s *t*-test was used to compare two groups when variables were quantitative, and Pearson’s chi-square or Fisher’s exact test, as appropriate, was used for qualitative variables. Paired tests were used to compare consecutive measurements within the same group. A *p*-value of 0.05 was considered the threshold for significance.

## 3. Results

A total of 77 patients were treated (35 males and 42 females), with the mean age being 57.27 ± 13.03 years (range 28–86 years). There were 11 smokers (14.3%). At the time of extraction, the FMBS value was equal to 20 in 1 case, greater than 20 in 30 cases, and less than 20 in 46 patients. The value of FMPS was greater than 40 in 20 cases and less than 40 in 57 cases.

Table 1 describes the outcomes; one post-surgical complication occurred (alveolitis) in the control group, and none was reported in the test group.

**Figure 1:**
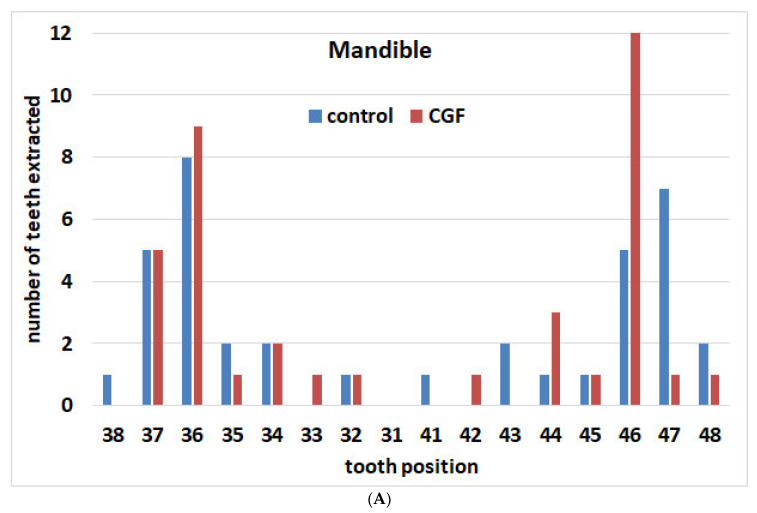

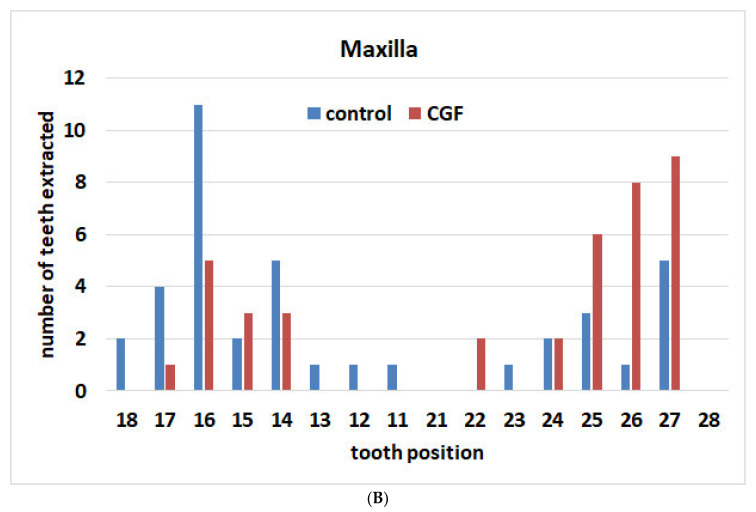
Distribution of extracted teeth according to location in the two groups. (**A**) Maxilla; (**B**) mandible.

The locations of the extracted teeth in the two groups are illustrated in Figure 1A,B. For the control group, in the maxilla, 4 anterior elements (from canine to canine) and 35 posterior elements (premolars and molars) were extracted. In the mandible, 4 elements were extracted in the anterior area, and 34 were in the posterior. For the CGF group, in the maxilla, 2 anterior elements and 37 posterior ones were extracted. In the mandible, 3 elements were extracted in the anterior area, and 35 were in the posterior.

The healing index on day 7 was significantly better (*p* < 0.001) in the test group (5.01 ± 1.30) than in the control group (6.65 ± 1.41).

**Figure 2:**
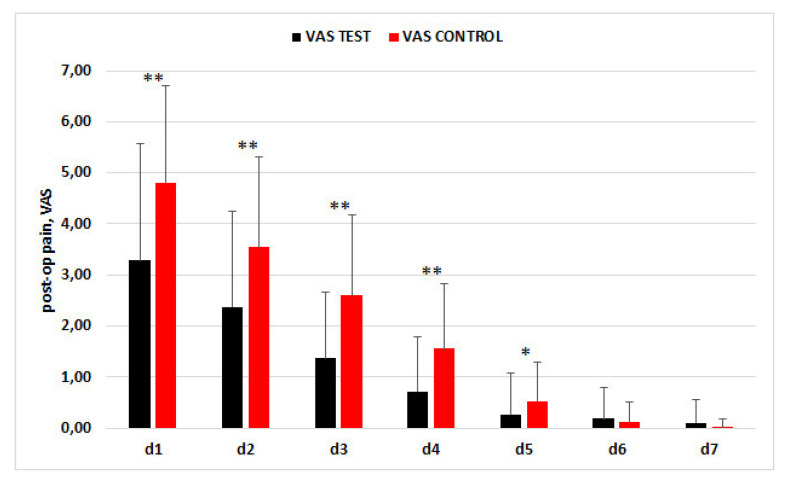
Trend of VAS scores in the two groups in the first week postextraction.* *p* < 0.05, ** *p* < 0.001.

The trend of the mean values for the VAS is presented in Figure 2. In the control group, the mean VAS score was significantly higher than in the CGF group until day 5 post-surgery.

**Figure 3:**
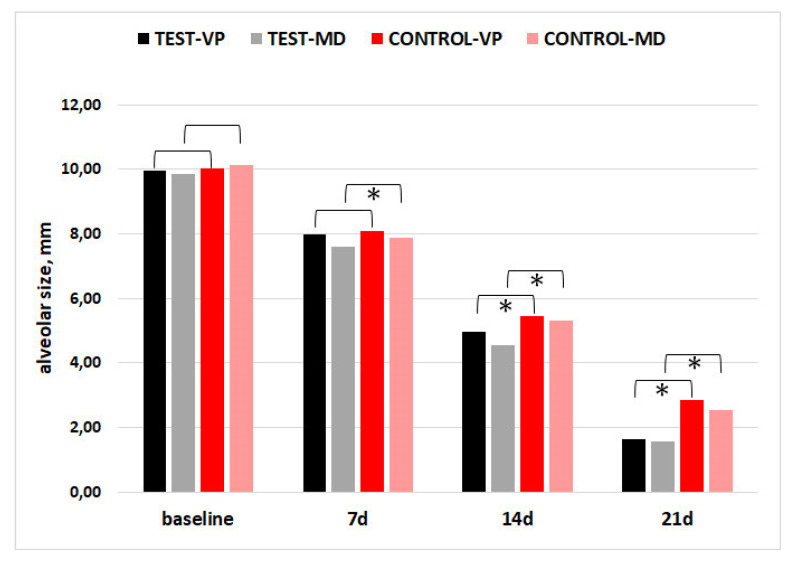
Alveolar socket size in the vestibulo-palatal/lingual (VP) and mesio-distal (MD) dimensions. Asterisks indicate significant differences between CGF and CTR groups (*p* < 0.05).

For alveolar socket dimensions (vestibulo-palatal/lingual, VP; mesio-distal, MD), none of the comparisons between groups at baseline showed significance. There was a trend toward lower alveolar dimensions in the CGF group, indicating faster healing, as compared to the control at 7, 14, and 21 days. In Figure 3, the asterisks indicate significant between-group differences.

**Figure 4:**
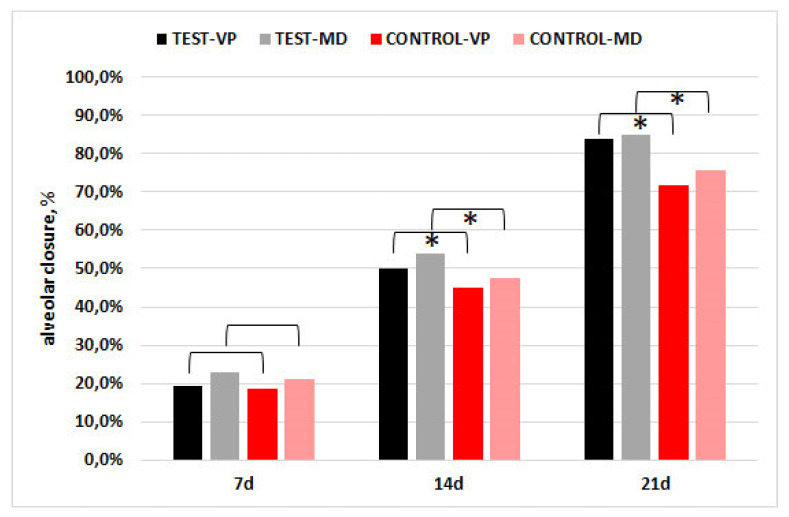
Percentage socket closure in the vestibulo-palatal/lingual (VP) and mesio-distal (MD) dimensions. The asterisks indicate that the difference between groups was significant (*p* < 0.001).

The socket closure % (vestibulo-palatal/lingual, VP; mesio-distal, MD) was greater in the CGF group relative to the control in both dimensions, and the difference was significant at 14 and 21 days (*p* < 0.001), as shown in Figure 4.

Five intraoperative complications were recorded in the group treated with CGFs, and three were reported in the other group (mostly in cases with root apex fracture). One postoperative complication (alveolar osteitis) was recorded in a control patient who had an intraoperative fracture of the root apex in the upper second left molar. No post-op complications occurred in the CGF group.

## 4. Discussion

A number of systematic reviews and evidence-based investigations have evaluated the effect of different types of autologous platelet concentrates in promoting postextraction socket healing and controlling side effects [1,8,12]. These studies have established that APCs have several advantageous properties compared to self-healing. The most commonly observed effect is soft tissue healing enhancement (better vascularization, faster epithelization, and accelerated closure of the socket as compared to control sites). Advantages for hard tissue healing have also been reported, assessed using different methods (biopsies undergoing histological and histomorphometric analysis, periapical radiographs, micro-CT, cone beam computed tomography, osseous scintigraphy, and clinical evaluation of changes in ridge height and width over time) [1,8,12]. Furthermore, less postextraction pain, fewer side effects, and reduced incidence of alveolar osteitis and other adverse events, as compared to control sites, were typical findings in clinical studies evaluating such outcomes. The best study design for our purpose was the split-mouth trial. In fact, this type of study allows for controlling the between-group variability that occurs in parallel trials due to differences in the healing process in response to treatment, in metabolic features, and in blood parameters among different subjects. The presence of a control group in which the sockets are left to heal spontaneously allows comparison with most of the earlier studies. The faster socket closure and reduced postoperative pain observed in the CGF-treated sites are in line with studies performed with different platelet concentrates, such as platelet-rich plasma (PRP), plasma rich in growth factors (PRGF), and platelet-rich fibrin (PRF) [1,8,12]. Nevertheless, one of the limitations of the present report is the lack of a direct comparison with another platelet concentrate, as this would have increased the value of the study. However, in the split-mouth design, the addition of a third group is challenging, as it is rather difficult to find an adequate sample of patients requiring the extraction of three or more comparable teeth in a reasonable amount of time.

Among the various platelet concentrates that are possible to obtain using current systems, L-PRF (leukocyte-rich PRF) is the most similar to CGFs. Concentrated growth factors, produced with the Medifuge System, were recently introduced in the field of APCs [9]. Similar to L-PRF, blood is collected in glass tubes without anticoagulant, but CGFs are obtained through a specific and standardized centrifugation procedure conducted at a steady temperature and at precisely controlled variable centrifugation forces. In addition, the rotation speed increases and decreases gradually at the beginning and at the end of the centrifugation step, respectively, in order to limit abrupt acceleration and deceleration, aiming to maximize cell integrity preservation.

Some in vitro investigations evaluated the characteristics and the biological activity of the CGF clot that was used in the present study and compared them to those of the advanced PRF clot (A-PRF) produced using the standard protocol [13,14]. Isobe et al., found that the cell composition, the mechanical strength, the kinetics of degradation, the thickness of fibrin fibers, and the crosslink density of CGF and A-PRF were very similar, despite considerable differences in the protocols of centrifugation [13]. In another in vitro study, Lee et al., found that CGFs have better mechanical properties (greater tensile strength) and higher amounts and concentrations of some growth factors (EGF and PDGF-BB) compared to PRF [14]. Furthermore, the proliferation of osteoblasts in cultures supplemented with CGF or PRF clots at variable proportions (5%, 10%, and 50%) was found to be equivalent to that of cultures grown in a medium supplemented with 10% FBS (fetal bovine serum). The osteoblast and gingival fibroblast numbers were significantly higher with CGFs as compared to PRF, independent of the preparation (10% and 50%) [14].

In a split-mouth study comparing L-PRF with spontaneous healing in postextraction sockets, Marenzi et al., used the same type of healing index that was used in our study and reported a mean value of 4.8 ± 0.6, which is close to the best healing score (5.0) and comparable to the findings of the present study [15]. Excellent scores of the original Landry healing index were reported in other studies that treated postextraction sockets with L-PRF [16,17,18,19]. Studies that investigated postextraction pain after treatment with L-PRF found VAS scores that are very similar to those observed in our study [15,16,17,18,19].

Based on the findings of in vitro investigations, the characteristics and biological activity of CGFs were highly comparable to those of PRF (13,14). Of course, the results of any in vitro study must be substantiated by clinical investigations.

To date, a limited number of studies have investigated whether the addition of CGFs has some beneficial effect on socket healing. This can be due to CGFs being more recently introduced as compared to other platelet concentrates. In a study published in 2019, Özveri Koyuncu et al., reported that CGFs produced significant benefits in soft tissue healing, postoperative pain, edema, and trismus compared to spontaneous healing after third molar surgery [20]. In a study on the management of alveolitis, Kamal et al., found that CGFs have a positive effect on wound healing acceleration and pain reduction compared to socket curettage and irrigation with saline solution [21]. Another trial by Kamal et al., compared three clinical approaches for the management of dry socket (low-level laser therapy (LLLT), CGF, and standard treatment, consisting of mild socket curettage plus irrigation with saline solution) [22]. They reported that the beneficial effects of CGF on pain reduction and healing rate were significantly greater than those of LLLT as compared to the control group. Such studies are difficult to compare to the present one because of different protocols; nevertheless, the reported benefits of CGFs for both pain relief and healing enhancement following tooth extraction are in agreement with our findings.

Very few clinical studies have compared the tissue healing response using CGFs and PRF. In a recent split-mouth study, Mozzati and coworkers evaluated the performance of CGFs vs. L-PRF (obtained with the Intraspin System^®^, Intra-Lock System Europa SpA, Salerno, Italy) in promoting postextraction socket healing using a protocol similar to the present one (23). Slightly better results were found for the pain score on day 1 and for socket closure on day 7 in favor of CGFs. No other clinical assessments showed differences between the two products [23].

Torul et al., in a three-group, parallel randomized study on mandibular third molar surgery, compared A-PRF, CGFs, and spontaneous healing [24]. Based on the VAS score, the number of painkillers taken, swelling, and trismus during the first week after surgery, they found that neither A-PRF nor CGFs had a significant advantage over the control in any of the outcomes investigated. That trial was difficult to compare to the one by Mozzati et al., for several reasons. First, Torul et al., did not assess the healing index or socket closure, which were the key quantitative outcomes of the Mozzati et al., study (and of the present study as well); second, Torul et al., performed a parallel study, while the one by Mozzati was a split-mouth trial. The results of the comparison of subjective outcomes, such as VAS scores, among different subjects belonging to different groups must be interpreted cautiously. In addition, A-PRF is produced using a different centrifugation system from the one that was used for L-PRF in the Mozzati et al., study. The latter, in fact, was found to be the most valuable among several centrifugation systems producing L-PRF [25], and its effectiveness in promoting tissue healing in many oral surgery procedures is well-documented [26].

## 5. Conclusions

CGFs represent an effective adjunctive option for safe and predictable healing of the postextraction socket in the early healing stage. Significantly lower VAS scores and the lack of post-surgical complications in the test group confirm the benefits of CGFs not only for promoting tissue healing but also for controlling patient discomfort, starting from the first day after surgery. Further comparative studies with histological and histomorphometric evaluation and with a longer follow-up are required to confirm the findings of the present study.

## Figures and Tables

**Table 1:** Main outcomes and characteristics of the groups. The significance of the between-group difference is shown in the right column.

	CGF	Control	*p*-Value
Maxilla/mandible (*n*. of elements)	39/38	39/38	*p* = 1.00
Cause of extraction (*n*. of elements)-Advanced caries	44	47	*p* = 0.86
-Periodontal disease	29	27	
-Tooth fracture	4	3	
Alveolar size VP/L at baseline, mean ± SD (mm)	9.95 ± 2.52	10.03 ± 2.66	*p* = 0.61
Alveolar size MD at baseline, mean ± SD (mm)	9.87 ± 3.38	10.14 ± 3.49	*p* = 0.10
Intraoperative complications (number)	5	3	*p* = 0.22
Postoperative complications (number)	0	1	*p* = 0.50
Healing index at 1 week (score)	5.01 ± 1.30	6.65 ± 1.41	*p* < 0.001

CGF = concentrated growth factor; VP/L = vestibulo-palatal/lingual; MD = mesio-distal; SD = standard deviation.

## Data Availability

All experimental data to support the findings of this study are available upon request by contacting the corresponding author.
